# Translation and psychometric evaluation of the reflective capacity scale in Iranian medical education

**DOI:** 10.1186/s12909-023-04791-3

**Published:** 2023-10-27

**Authors:** Zohreh Khoshgoftar, Maasoumeh Barkhordari-Sharifabad

**Affiliations:** 1 School of Medical Education and Learning Technologies, Shahid Beheshti University of Medical Sciences, Tehran, Iran; 2 School of Medical Sciences, Yazd Branch, Islamic Azad University, Yazd, Iran

**Keywords:** Psychometric validation, Scale, Reflection, Medical education, Reflective capacity, Students

## Abstract

**Background:**

Examining the reflective capacity in medical students is a principal step for the development of effective educational strategies to improve it. Reflection scales available in Iran are inadequate due to the lack of focus on students’ willingness and tendency to participate in reflection. This study aimed at translation and psychometric evaluation of “Reflective Capacity Scale” in Iranian medical education.

**Methods:**

This methodological research was completed in two parts: translation and psychometric evaluation. After getting permission from the main developer of the tool, the translation process was done based on Polit and Yang model in Persian language. Then, face validity and content validity of the tool were established using a qualitative method. Construct validity was surveyed with exploratory and confirmatory factor analysis via completing the questionnaire by 320 medical students, who were selected using convenience sampling. The reliability of the tool was also checked with two methods of internal consistency and stability. The gleaned data were analyzed with SPSS20 and AMOS.

**Results:**

“Reflective Capacity Scale” includes 16 items that were retained after cross-cultural translation. Face validity and content validity were acceptable. By performing exploratory factor analysis, four factors were identified that accounted for 63.79% of the total variance. In the confirmatory factor analysis, the values of the fit indices confirmed the appropriate fit of the model. The internal consistency reliability of the whole tool was equal to 0.83 and the intra-class correlation coefficient was equal to 0.98.

**Conclusion:**

The translated and validated “Reflective Capacity Scale” provides a robust tool for assessing reflective capacity among Iranian medical students. Its validity and reliability underscore its potential for measuring the reflective capacity of medical students.

## Background

Reflection is an indispensible component of learning in medicine [1, 2], which can promote competence, humanism, and professionalism by developing self-regulated learning skills [1, 3, 4]. It is considered an instrument to advance knowledge, guide future learning, deepen understanding of complex concepts, and explore emotionally challenging situations [1, 5, 6]. The necessity of these cases is more felt in contemporary medicine because contemporary medicine deals more than ever with complex challenges pertaining to aging, chronic diseases, comorbidity, etc. [7].

Reflective capacity refers to “students’ ability, willingness, and inclination to participate in reflective thinking during their studies and clinical practices” [8]. This capacity is an important ability that allows doctors to be alert, interested, aware and ready to identify and correct errors [9]. Given the importance of reflective practice for medical education, it is important to develop valid and reliable tools to assess the capacity and ability of reflective thinking [8]. The existence of such tools makes it possible to evaluate interventions related to reflective capacity and the impact of reflective capacity on learning and performance [10].

Medical lecturers/instructors can check the capacity of students to reflect through the analysis of the process of “problem solving” and “clinical decision-making” [11, 12]. Some instruments in this field allow the evaluation of levels of reflection and guided feedback [13–16]. There are also tools that examine reflection for specific disciplines such as medical professionals [17] and pediatric mental health professionals [18]. One of the most well-known reflective thinking scales was developed by Kember et al. to explore different dimensions of reflective thinking (habitual action, understanding, reflection and critical reflection) [19]. Most of the available questionnaires evaluate the skill of reflection, but for any skill, it can be said that a skilled person has the ability to perform that skill, even if s/he does not use that skill at that moment. What most experts agree on is that individual characteristics, mental habits, attitudes, or emotional tendencies should also be examined in the evaluation of any skill.

Reflective Practice Questionnaire (RPQ) is one of the questionnaires developed by Priddis and Rogers in 2018. This questionnaire can be used in a wide range of fields and professions, as well as in many types of activities in which services are provided to people. Service recipients in these interactions can be clients, patients, customers, students, or any other term that a profession uses to describe its service recipients [20]. RPQ has been used in the field of medical education and it has been rendered as a reliable tool for evaluating reflective capacity and its related characteristics in medical students [8]. Priddis and Rogers (2018) suggest that RPQ subscales may be used selectively depending on practical limitations and goals [20]. The Reflective Capacity Scale (RCS) is a subscale of the RPQ that has four dimensions of reflection during performance, reflection after performance, reflection with others, and active self-evaluation [8, 20].

Since the introduction of the RPQ, various studies have used this scale to explore different dimensions of reflective performance. The complete RPQ has been used in medical education in the United States [8] and to assess the reflective capacity of medical students [21, 22]. Also, the validity and reliability of the Swedish version of the reflective capacity subscale in nursing education has been confirmed [10].

Despite the high reliability and validity of this questionnaire in different contexts, it is generally recommended that the scale be contextualized in each cultural context to ensure its validity. The tools that exist to measure reflection in Iranian medical education, such as Rubik’s Reflection Evaluation for Learners’ Enhanced Competencies Tool (REFLECT) and Kember’s Reflection Thinking Scale, examine the levels of reflection. However, it is also important to examine the desire and tendency of students to participate in reflection. This study was conducted with the aim of translation, cultural adaption, and psychometric evaluation of the “Reflective Capacity Scale” to be used both in research and practice because there was no such instrument found in Iran to examine the reflective capacity of medical students, and on the other hand, the implementation of interventions for the development of reflective capacity in medical education requires the existence of a valid and reliable measurement instrument to assess reflective capacity. The product of this research is directly applicable to medical students, professors, and the medical education system of Iran. Moreover, with the improvement of education, the patients and clients of the health system also may benefit indirectly.

## Methods

### Study design

This cross-sectional descriptive study evaluated the cultural compatibility and psychometric properties of the Reflective Capacity Scale in Iranian medical education.

### Research sample

The research population consisted of medical students studying in Shahid Beheshti University of Medical Sciences, Tehran/Iran. Participants were selected from July 2022 to September 2022 using convenience sampling. The inclusion criteria for participating in the study were: interns studying in the field of medicine, and providing informed consent to participate in the study. The number of samples in the translation stage included two translators familiar with Persian and English in forward translation and two translators in back-translation. To determine face validity, 10 medical students were selected and to determine content validity, 10 experts were selected using purposive sampling method [23]. Factor analysis complies with the general rule of sampling knowledge that the number of subjects ought to be always more than the number of variables. To determine construct validity, 5–10 people are needed for each item of the instrument [24]. In this research, 162 medical students were selected for exploratory factor analysis and 158 for confirmatory factor analysis. Between 15 and 20 samples are recommended in establishing the reliability of the tool [25]. Thus, 20 samples were used in this research.

### Translation and Psychometric Evaluation of the Tool

#### 1. Translation stage

After obtaining permission from the original developer, the Reflective Capacity Scale was translated from English to Persian following the translation guidelines by Polit and Yang [26]. In the first stage, the translation of the tool from English to Persian was done separately by two Iranian translators who were fluent in Persian and English languages and cultures. Subsequently, Persian translations were reviewed with the presence of experts to create a single translation. In the next stage, the Persian translation was repeated again by two other translators, fluent in both Persian and English languages, without knowing the main items of the tool, and then, with the consultation and opinion of experts, the version translated into English was agreed upon. Finally, the final revised version was sent to the main developer of the tool for feedback, which was approved.

#### 2. Psychometric stage

Next, the tool translated into Persian was given to 10 students to determine the face validity using a qualitative method, and the items were examined in terms of difficulty level, ambiguity, and appropriateness [27]. In the next step, to evaluate the content validity, 10 experts in medical education, reflection and psychometrics were asked to give their professional subjective judgment and viewpoints on the relevance, necessity, representativeness, and comprehensiveness of the items. In the present study, construct validity was investigated using exploratory and confirmatory factor analysis. Then, the reliability was examined by the method of internal consistency and stability (Cronbach’s α coefficient). To check the stability reliability, the Persian version of the scale was completed with an interval of 2 weeks.

### Data collection tool

In this research, the following tools were used to collect data:

1. Demographic Information Questionnaire: This questionnaire was applied to obtain personal information in areas such as age, gender, grand point average, and marital status.
2. “Reflective Capacity Scale”: This scale is a subscale of the Reflective Practice Scale developed by Priddis and Rogers in 2018. This scale has four dimensions of reflection during performance, reflection after performance, reflection with others, and active self-evaluation (3, 32). Items 4, 7, 11, & 14 are related to the dimension of reflection during performance, items 2, 8, 10, & 13 are related to the dimension of reflection after performance, items 1, 5, 12, & 16 are related to the dimension of reflection with others, and items 3, 6, 9 & 15 are related to the dimension of active self-evaluation. All items in the scale are scored based on a 6-point Likert scale: (1) not at all, (2) slightly, (3) somewhat, (4) moderately, (5) very much, (6) extremely. In this way, the grades range from 1 to 6. A higher score indicates a greater capacity for reflection. Cronbach’s alpha was 0.84 for overall scale [8].

### Data analysis

The Kaiser-Meyer-Olkin (KMO)’s measure of sampling adequacy and Bartlett’s test of Sphericity were used to determine the factor ability of the sample and the fit of the factor analysis. A KMO value higher than 0.5 is acceptable [28, 29]. EFA was performed by principal component analysis followed by varimax rotation. Eigenvalues and factor loadings were considered higher than 1 and 0.3, respectively [30]. Then, the confirmatory factor analysis method was used to confirm the dimensions of the questionnaire and the proposed model of exploratory factor analysis. In this study, indices of Chi-square, Root Mean Square Error of Approximation (RMSEA), Normed Fit Index (NFI), Goodness of fit index (GFI), and Adjusted goodness of fit index (AGFI) were evaluated. In the reliability check, the obtained scores were compared with the intra-class correlation test. Cronbach’s α and ICC values higher than 0.7 are considered satisfactory for interpreting the results [31].

## Findings

Totally, among the 325 questionnaires received, 320 questionnaires were fully completed and 5 questionnaires were excluded due to incomplete answers. Based on the findings presented in Table 1, the average age of the participants was 24.75 ± 1.85 years and their average GPA was 16.61 ± 1.13. The majority of participants were female (50.93%) and single (88.43%) (Table 1).

**Table 1:** Demographic characteristics of the participants

Variables		Mean ± SD	N (%)
Age		24.75 ± 1.85	
Grand point average (GPA)		16.61 ± 1.13	
Gender	Male		157 (49.06)
	Female		163 (50.93)
Marital status	Married		37 (11.56)
	Single		283 (88.43)

### Face validity results

The findings from the examination of students’ opinions about each item of the scale showed that all the items were understandable for the students and there were no ambiguous items; so, no changes were made to the items at this stage. In general, it seems that “the Reflective Capacity Scale for Medical Education” is not much different from the cultural content of Iranian students. Therefore, the Persian version of this scale was evaluated as conceptually clear, appropriate, and satisfactory.

### Content validity results

In terms of qualitative content validity, the suggestions of experts were applied in terms of the relevance of items with the intended concept and the use of appropriate diction and wording, the placement of phrases in the appropriate place. The experts believed that all the items are related to the intended target and topic, and also necessary and suitable for the assessment of the reflective capacity of medical students. They had given suggestions in terms of correction of grammar and sentence patterns. Finally, the items were modified and beautified as per experts’ suggestion keeping in mind the same content of those items. The modified items were item number 8, 9, and 11.

### Construct validity results

KMO was 0.87 and significant, which means that the data were suitable for performing factor analysis. Besides, the value of Bartlett’s sphericity test index was 1115.88/120 and the correlation matrix between items was significant (P < 0.001), which indicated detectible relationships between variables.

**Fig. 1:**
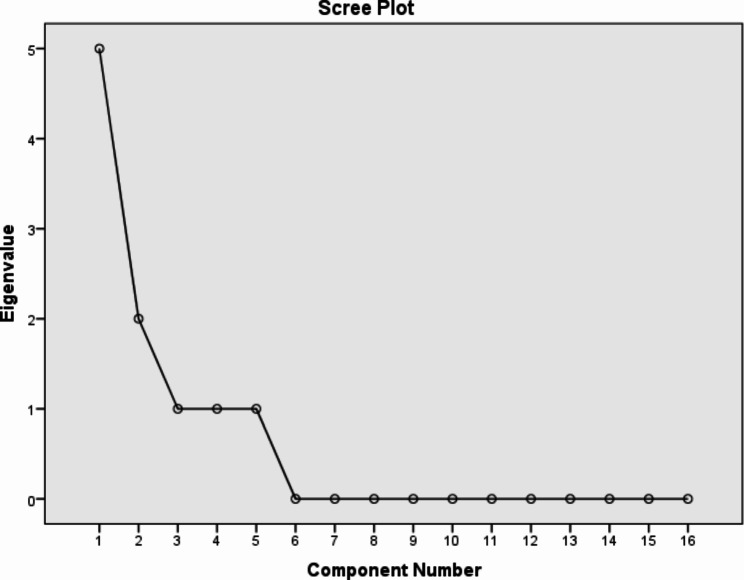
Scree plot determining the number of suitable factors that can be extracted

First, in the exploratory factor analysis with varimax rotation, 5 factors with eigenvalues greater than 1 were obtained, the results of which are shown in Table 2; Fig. 1. The factor loading of all the items was more than 0.3 and none of the items were deleted. According to Table 2, in factor five, only one item was obtained, and considering that the factor load of this item was acceptable in factor 1 and according to the main scale, this item was integrated into this factor and finally 4 categories were obtained so that 69.73% of the variance was accounted for by the 4 factors.

**Table 2:** Items and factor loading related to the extracted factors

	Items	Factor Structure				
		1	2	3	4	5
1	When I reflect on my work with others, I become aware of matters that I had not considered before	0.76				
5	When I reflect on my work with others, I come to new perspectives.	0.67				
12	I find reflecting with others about my work helps me to solve the problems I may face.	0.63				
9	I think about how I can improve my ability to work with clients.		0.75			
6	I think about my weaknesses in working with clients.		0.75			
15	I critically evaluate the strategies and techniques I use in working with clients.		0.71			
3	I think about my strengths in working with clients.		0.68			
2	After interacting with clients, I spend time thinking about what happened.			0.74		
10	After interacting with clients, I think about my experience from this interaction.			0.66		
13	After interacting with clients, I reflect on how things went during the interaction.			0.65		
8	After interacting with clients, I think about the client’s experience of this interaction.			0.64		
4	During interacting with clients, I recognize times when my prior beliefs influence the interaction.				0.91	
14	During interactions with clients, I consider how their (clients’) personal thoughts and feelings affect the interaction.				0.83	
11	During interacting with clients, I recognize when the client’s previous beliefs affect the interaction.				0.75	
7	During interactions with clients, I consider how my own thoughts and feelings affect the interaction.				0.64	
16	When I reflect with others about my work, I gain new insights.					0.80
**Explained variance**		5.53	2.11	1.38	1.19	1.07
**Explained %**		34.55	13.17	8.60	7.47	6.68
**Cumulative %**		34.55	47.72	56.32	63.79	70.47

According to the results obtained from the exploratory factor analysis, four main factors called: reflection during performance (items 4, 14, 11, 7), reflection after performance (items 2, 10, 13, 8), reflection with others (items 1, 5, 12, 16), and active self-evaluation (items 9, 6, 15, 3) were extracted.

Confirmatory factor analysis was used to check the construct and confirm the dimensions of the questionnaire. In this research, confirmatory factor analysis was performed with the help of AMOS. The values of fit indices in the confirmatory factor analysis indicated the appropriate fit of the model (Table 3).

**Table 3:** Goodness of fit indices

Indices	Observed value	Acceptable fit
*χ* ^*2*^	114.1	
Df	98	
*χ*^*2*^/df	1.16	< 2
P. value	0.9	> 0.05
GFI	0.93	> 0.90
AGFI	0.95	> 0.90
RMSEA	0.03	< 0.05
NFI	0.97	> 0.90

*χ*^*2*^: Chi-square, df: Degrees of Freedom, RMSEA: Root Mean Square Error of Approximation, NFI: Normed Fit Index, GFI: Goodness of fit index, AGFI: Adjusted goodness of fit index

**Fig. 2:**
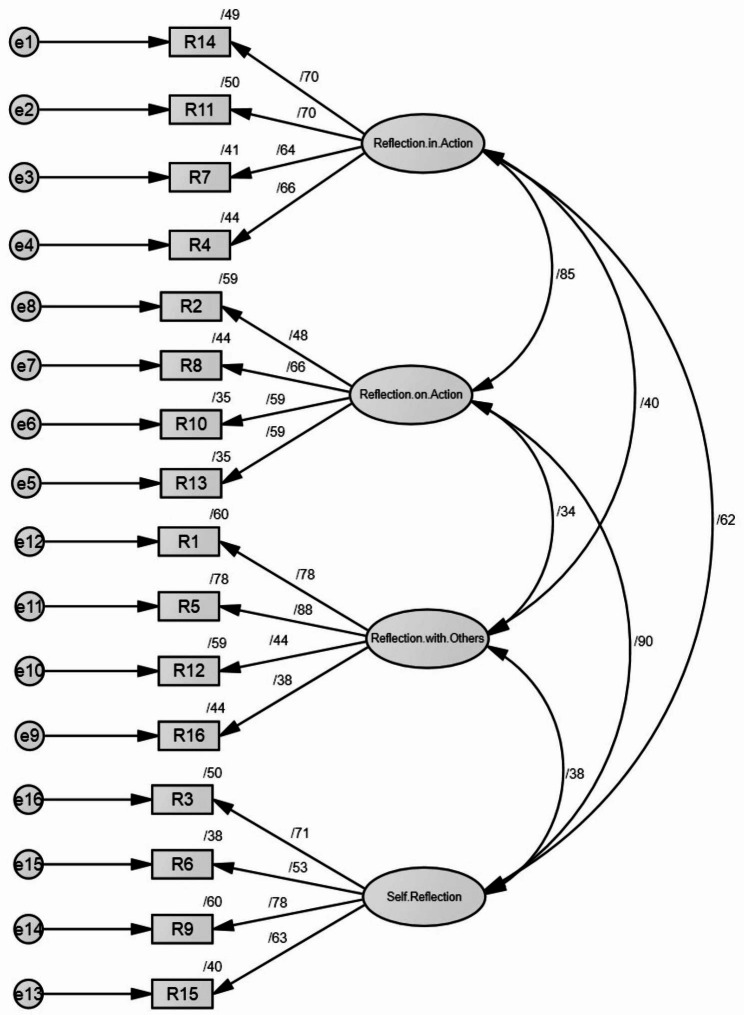
Results of confirmatory factor analysis

The results of the confirmatory factor analysis based on the factor statistics of the model showed that the factor loading of all indicators and components was above 0.3; therefore, the membership of all investigated factors in this variable has been confirmed (Fig. 2).

### Reliability establishment results

To determine the reliability, internal consistency method and intra-class correlation coefficient (ICC) were used. Cronbach’s α coefficient was equal to 0.83. All subscales had Cronbach’s alpha values more than 0.70, that indicates had a satisfactory internal correlation (Table 4).

The ICC was 0.98, which is favorable. This coefficient for dimensions of reflection during performance, reflection after performance, reflection with others, and active self-evaluation were 0.93, 0.93, 0.83, and 0.94 respectively (Table 4).

**Table 4:** **Cronbach’s alpha and ICC for reliability of** the Reflective Capacity Scale **(Persian version)**

Factors	Number of items	Cronbach’s alpha	ICC
Reflection during performance	4 items (4, 7, 11, 14)	0.76	0.93
Reflection after performance	4 items (2, 8, 10, 13)	0.73	0.93
Reflection with others	4 items (1, 5, 12, 16)	0.79	0.83
Active self-evaluation	4 items (3, 6, 9, 15)	0.76	0.94
Total	16 items	0.83	0.98

## Discussion

This research aimed at translating and psychometrically evaluating the Reflective Capacity Scale in medical education in Iran. The scores obtained using this scale can be an indicator for evaluating educational interventions with the aim of improving the reflective capacity of medical students [10]. This assumption and the lack of a valid and reliable tool to measure the reflective capacity of Iranian medical students motivated the decision for translation, cross-cultural adaptation and its validation.

The findings of the translation phase in this research suggested the acceptability of the translation of the original scale into Persian. Although there have been different guidelines for cross-cultural adaptation [32], the forward and backward translations used in this study have been implemented in previous research [10]. In the current study, the seven-step model proposed by Polit and Yang [26] was used, and the utilization of this model in various research shows its importance and acceptability [33, 34]. Translation and cultural adaptation of existing tools, instead of developing new tools, provides the possibility of extracting comparable data using valid questionnaires and facilitates the exchange of information among scientific community [35].

After completing the translation process, it is necessary to check and determine the validity and reliability of the questionnaire in the target population [32]. Due to the importance of the understanding of the items by the target group, especially in instruments that are developed for a specific population, face validity is considered an important step in validity analysis [36]. In determining the face validity, all of the items in the Persian version of the scale were well understood by the students.

The results of the content validity investigation indicated approval of the content validity of the scale. In the present study, content validity was evaluated by 10 experts and the items were edited according to the experts’ recommendations. The process of examining the clarity and content equivalence gave more support to the conceptual, semantic, and content equivalency as well as the structure of sentences used in the translated version [37, 38].

The results of factor analysis were 16 items that were placed in four dimensions and accounted for 63.79% of the total variance. Naming factors is a subjective process wherein theoretical concepts are usually used [39]. To label the four dimensions proposed by factor analysis, the original scale developed by Priddis and Rogers, as well as the meaning of the items of each dimension alone and in relation to other items [20] were considered; subsequently, the four factors were classified as: “reflection during performance”, “reflection after performance”, “reflection with others”, and “active self-evaluation”. The results of the confirmatory factor analysis also showed that the values of the fit indices indicated the acceptable fit of the proposed model with the data. Of course, the Swedish version of this scale was a single-factor one [10].

“Reflective thinking during performance” involves considering the prior beliefs, thoughts, and feelings of the individual and the client during the interaction that can influence the interaction. “Reflection after the performance” is related to the interaction with the client and reflection on what was said and done [20]. Reflection subscales during performance and after performance are concepts that are also mentioned by Schon [40]. “Reflecting with others” includes issues such as gaining new awareness, perspective, and insight when examining the process of interaction and performance with others. Ultimately, “active self-evaluation” is reflecting about strengths and weaknesses when working with clients, improving abilities and critically evaluating strategies and techniques used when working with clients [20].

Reliability of the tool is one of the most important criteria that discloses the quality of the tool. A Cronbach’s alpha value of 0.83 was obtained in the internal consistency analysis. Cronbach’s α in the Swedish version of this scale was 0.91 [10]. In Rogers et al.‘s study, Cronbach’s α was 0.84 [8]. In the reliability analysis through stability, the ICC of 0.98. According to the results, it can be claimed that the Persian version of the scale has good stability.

### Implications for medical education

The validation process of the “Reflective Capacity Scale” confirmed its use in Iranian medical education. Since fostering reflection can improve many aspects of medical education, including professional development and patient-centered care [41], this scale can be used as part of evaluating interventions related to reflective capacity and the impact of reflective capacity on learning and performance [42]. Persian version of the scale can help to raise the awareness of trainers, managers and policy makers of medical education about the reflective capacity of Iranian medical students and plan accordingly to increase this capacity. By taking the necessary measures to improve the level of students’ reflective capacity, it is possible to help implement the mission of medical education in promoting patient-centered care and increasing the health level of Iranian society.

### Limitations of the study

Due to the fact that this tool was a new one that was developed in English and translated and validated only in Swedish, therefore, the researcher faced some problems in obtaining pertinent literature in this field for a better discussion. The convergent and discriminant validity were not investigated. In this research, due to the small number of items and the fact that scale content area was already specified, the quantitative phase was not conducted to calculate the content validity index (CVI) and content validity ratio (CVR). Additionally, the mentioned scale is a self-report measure that may be associated with social desirability bias. In this study, only college students studying in one university were studied using convenience sampling; hence, caution should be exercised in generalizing the results. It is worthwhile to conduct future studies in several provinces and cities at a national level with a significant sample size.

## Conclusion

This study confirmed that the Persian version of the “Reflective Capacity Scale” is a reliable and valid instrument to evaluate reflective capacity in medical students within Iran. The structure of the dimensions obtained in this study was consistent with the structure of the original scale, including “reflection during performance”, “reflection after performance”, “reflection with others”, and “active self-evaluation”. The psychometric validation indicated that the Persian version of the scale has satisfactory reliability. The application of this tool is easily possible and can be completed by people in a short time.

## Data Availability

The datasets generated and analyzed during the current study are not publicly available due to an agreement with the participants on the confidentiality of the data but are available from the corresponding author on reasonable request.

## List of abbreviations

RPQ: Reflective Practice Questionnaire
RCS: Reflective Capacity Scale
REFLECT: Reflection Evaluation for Learners’ Enhanced Competencies Tool
χ^2^: Chi-square
df: Degrees of Freedom
RMSEA: Root Mean Square Error of Approximation
NFI: Normed Fit Index
GFI: Goodness of fit index
AGFI: Adjusted goodness of fit index
SD: Standard Deviation
KS: Kolmogorov-Smirnov
GPA: Grand point average
KMO: Kaiser-Meyer-Olkin
CVI: Content Validity Index
CVR: Content Validity Ratio
